# Combined effects of air pollutant mixtures on Alzheimer's disease‐related cortical atrophy

**DOI:** 10.1002/alz70860_096366

**Published:** 2025-12-23

**Authors:** Heeseon Jang, Hyunji Park, Hyunah Son, Changsoo Kim

**Affiliations:** ^1^ Yonsei University College of Medicine, Seodaemun‐gu, Seoul, Korea, Republic of (South)

## Abstract

**Background:**

Little is known about the combined effects of various air pollutants on Alzheimer's disease (AD)‐specific brain structural pathologies. Hence, we aimed to examine the associations of air pollutant mixtures with subclinical brain imaging AD markers in dementia‐free adults.

**Method:**

This cross‐sectional study included 649 dementia‐free adults (≥50 years), dwelling in two metropolitan and two small cities in the Republic of Korea. All participants underwent brain magnetic resonance imaging (MRI) scans. The five‐year average concentrations before enrollment of particulate matter with diameters ≤ 10 μm (PM_10_) and ≤ 2.5 μm (PM_2.5_), NO_2_, Mn in PM_10_ (Mn_10_), and volatile organic compounds (acetaldehyde, styrene, and toluene) were estimated at each participant's residential address. AD‐related subclinical imaging markers, including the AD‐specific cortical atrophy score and whole‐brain cortical thickness, were derived from 3‐T brain MRI scans. We performed Bayesian kernel machine regression (BKMR) to examine combined effects of seven air pollutant mixtures on AD‐related subclinical imaging markers.

**Result:**

The BKMR model showed that the overall effect of air pollutant mixtures on AD‐specific cortical atrophy scores was significantly lower at the 25th percentile compared with that at the 50th percentile, whereas whole‐brain cortical thickness was significantly greater. We also found that NO_2_ contributed the most to subclinical imaging markers within the mixture.

**Conclusion:**

The findings suggest that air pollutant mixtures may induce AD‐specific cortical atrophy in dementia‐free adults.

